# Experimental design approach to the optimization of PAHs bioremediation from artificially contaminated soil: application of variables screening development

**DOI:** 10.1186/s40201-015-0178-y

**Published:** 2015-03-20

**Authors:** Masoumeh Ravanipour, Roshanak Rezaei Kalantary, Anoushiravan Mohseni-Bandpi, Ali Esrafili, Mahdi Farzadkia, Samireh Hashemi-Najafabadi

**Affiliations:** Department of Environmental Health Engineering, School of Public Health, Tehran University of Medical Sciences, Tehran, Iran; Department of Environmental Health Engineering, School of Public Health, Iran University of Medical Sciences, Tehran, Iran; Department of Environmental Health Engineering, School of Public Health, Shahid Beheshti University of Medical Sciences, Tehran, Iran; Department of Chemical Engineering, Biotechnology Group, Tarbiat Modares University, Tehran, Iran

**Keywords:** Bioremediation, Polycyclic Aromatic Hydrocarbons, Response Surface Method, Nutrient, Tween80

## Abstract

**Background:**

The effectiveness of bioremediation systems for PAH-contaminated soil may be constrained by physicochemical properties of contaminants and environmental factors. Information on what is the most effective factor in bioremediation process is essential in the decision of what stimulations can be taken to assist the biodegradation efficacy.

**Methods:**

In this study, four factors of surfactant (Tween 80), humic acid (HA), salinity and nutrients in a 2^4^ full factorial design were screened in bioremediation of phenanthrene contaminated soil by using a consortium of bacteria.

**Results:**

Between the employed levels of the factors only salinity had not significant effect. Optimal concentrations of surfactant, HA and nutrient were obtained by a response surface design. For phenanthrene biodegradation, a central composite face centred design (CCFD) showed that nutrient, surfactant and HA concentrations had highly significant, significant and insignificant effects, respectively. The best conditions with 87.1% phenanthrene biodegradation were 150 mg HA/Kg soil, 12.68 μg/L surfactant, and nutrients as K_2_HPO_4_, 0.8; KH_2_PO_4_, 0.2 and KNO_3_, 1 g/L. A high similarity was between the model prediction and experimental results.

**Conclusions:**

This study showed that nutrient with 81.27% efficiency could be considered as the most effective factor for practical implications of bioremediation process for PAHs contaminated soil cleanup strategies.

## Background

Petroleum derivatives are introduced into the environment through different ways such as anthropogenic activities, incomplete combustion of petroleum products, wood and coal, undesirable discharging of oil tankers, spills around petroleum refineries and gas plant facilities [1]. Polycyclic Aromatic Hydrocarbons (PAHs) are a group of these compounds with carcinogenic and toxic potentially [2,3] which contribute to environmental contamination and health hazards. Soil and sediments are the most important environmental reservoir for PAHs.

At present, employing biological treatment is the most popular and cost – effective strategy among the different methods to remove these pollutants from the soil [4]. However, successful application of bioremediation is often limited by environmental, physical and chemical factors [5] such as availability of pollutants to undergo biological transformations [6] toxicity and complex structures of PAHs derivatives, limitation for nitrogen, phosphorus or other nutrients, pH and temperature [7].

One of the main factors limiting bioavailability is the low aqueous solubility of PAHs. Addition of surfactant can be used for increasing the phase partitioning of organic compounds and their bioavailability. On the other hand bioavailability may be decreased by up taking of contaminants into the surfactant micelle [8]. Thus, the concentration of surfactant plays an important role in biodegradation of PAHs.

Many studies have been conducted to overcome problems related to the poor bioavailability of PAHs by using organic matter. Borresen and Rike [9] have shown that humic substances (HS) can increase the solubility of PAHs leads in increasing the bioavailability of PAHs in soil.

Nutrient such as nitrogen and phosphorus are the other important factors on biodegradation. Microorganisms need for nutrients similar to their composition of cells [10,11]. Betancur-Galvis LA et al. [12] used biosolid and inorganic fertilizer in bioremediation of phenanthrene.They found that the removal of phenanthrene in the soil with fertilization was 25 fold more than the other soils. There is a relation between mineralization rates of phenanthrene and the initial concentrations of nitrogen and phosphorus in the soils [13].

The marine soil and sediment is one of the most petroleum contaminants sites, so the salinity is an important factor which must be investigated. Minai-Tehrani D. et al. [14] showed that increasing salinity content of soil had decreasing effect on the biodegradation of total crude oil and PAHs.

The bioremediation strategy is dependent on the optimizing the factors which affect on the microbial growth and biodegradation of pollutant [15]. In combination of several factors, the effect of anyone may be influenced by the others and interactions among them may be occurred too.

The experimental design can be used for optimizing operational conditions for the multivariable system [16] and the interaction between variables would be considered too [17]. The number of experiment’s runs would be reduced by statistical design of experiments [17,18]. In this study numerous factors, involving surfactant (Tween 80 (Su.)), salinity (Sal.), soil nutrients (N, P (Nu.)) and organic matters (humic acid (HA)), individually and in combination, have been studied to remedy PAH artificially contaminated soils. The aim of this study was to investigate the effect of physicochemical factors; HA, surfactant (Tween 80), salinity and nutrient (N, P) together on the bioremediation of phenanthrene a three-ring PAH, an appropriate model compound, in soil slurry. In order to find out the most effective factor and the sequence importance of them in biodegradation of phenanthrene a three-ring PAH in the soil slurry, the study was conducted in two phase: 1) Screening the factors by using full factorial experimental design, and 2) Optimization of the phenanthrene-contaminated soil biodegradation by using a central composite face centered design (CCFD) under response surface methodology (RSM). Then the optimized condition was examined in PAHs real contaminated soil.

## Methods

### Chemicals

Acetone in HPLC grade was purchased from ROMIL Company. Phenanthrene (Purity > 98%), NaCl and chemical materials for mineral salt medium (MSM) were purchased from Merck Company. HA and Tween80 were supplied by Sigma Aldridge and Fluka, respectively. The formula of HA was C_17_H_17_BrN_2_O_5_. Nutrient Broth and R2A Agar were purchased from BIOMARK Company.

### Phenanthrene biodegradation

Clean soil was collected from a depth of 5–20 cm of ground’s surface, Tehran, Iran. It was air dried and passed through a 2-mm sieve. The soil was classified as sand (consisted of 89% sand, 11.9% silt and 5% clay) by the use of standard sieves. Total nitrogen and phosphorus were 0.025% and 0.0012%, respectively. Total organic carbon was 0.18%. The pH and electrical conductivity (EC) were 7.4 and 3.2 ds/m, respectively.

Two grams of dry soil was placed into 50 mL Erlenmeyer flask as non-continuous bioreactors. The bioreactors containing clean soil were autoclaved. Then, it was spiked with dissolved phenanthrene in acetone to have 500 mg phenanthrene/kg dry soil. The bioreactors containing spiked soil were placed in a shaker (Heidolph, ProMax 2020 model) at the velocity of 180 rpm in room temperature and dark condition to have a uniform dispersion of phenanthrene and evaporation of acetone.

The soil was inoculated with a consortium of bacteria in different concentration of MSM with an optical density of 1 at 630 nm [5] using CECIL UV/Vis spectrophotometer (model 7100). The bacterial consortium was consisted of *Bacillus sporogenes*, *Bacillus licheniformis*, *Capnocytophaga ochracea* (presumably), *Acinetobacter sporogenes* and *Staphylococcus xylosus*. Enrichment of the consortium and the potential of it in bioremediation of phenanthrene contaminated soils had been proved in our previous study [6]. The base of MSM was contained of the following (per liter): 0.2 g MgSO_4_.7H_2_O, 0.1 g CaCl_2_.2H_2_O, 0.1 g NaCl, 0.01 g FeCl_3_.6H_2_O and 1 mL trace element solution. The trace element solution contained the following (per liter): 23 mg MnCl_2_.2H_2_O, 30 mg MnCl_4_, 32 mg H_3_BO_3_, 39 mg CoCl_2_.2H_2_O, 50 mg ZnCl_2_, 30 mg NaMnO_4_.2H_2_O and 20 mg NiCl_2_ [5]. Then the amount of K_2_HPO_4_, KH_2_PO_4_, KNO_3_, HA, Surfactant and NaCl were added according to Tables 1 and 2 for phase 1 and 2 respectively. The pH was adjusted to 6.8 ± 0.2 using a pH meter (HACH 40d model). At the end, the slurry of soil liquid ratio was 10% w: v [6]. All the samples and their similar blanks were put in the shaker at the velocity of 180 rpm in room temperature (22 ± 3°C) for 8 weeks.

**Table 1:** **Actual values coded and of variables used in the full factorial (2** ^**4**^ **) design**

**Run**	**Actual value/coded Levels**					
	**Salinity (Sal.)%W/V**	**Surfactant (Su.)L/Lμ**	**Humic Acid (HA)mg/Kg**	**Nutrient(N, P)( Nu.) g/L**		
				**KNO**_**3**_ **KH**_**2**_ **PO**_**4**_ **K**_**2**_ **HPO**_**4**_		
R1	0 (−1)	0 (−1)	0 (−1)	0.000132 (−1)	0.000103 (−1)	0.0017 (−1)
R2	200 (1)	0 (−1)	0 (−1)	0.000132 (−1)	0.000103 (−1)	0.0017 (−1)
R3	0 (−1)	13 (1)	0 (−1)	0.000132 (−1)	0.000103 (−1)	0.0017 (−1)
R4	200 (1)	13 (1)	0 (−1)	0.000132 (−1)	0.000103 (−1)	0.0017 (−1)
R5	0 (−1)	0 (−1)	2 (1)	0.000132 (−1)	0.000103 (−1)	0.0017 (−1)
R6	200 (1)	0 (−1)	2 (1)	0.000132 (−1)	0.000103 (−1)	0.0017 (−1)
R7	0 (−1)	13 (1)	2 (1)	0.000132 (−1)	0.000103 (−1)	0.0017 (−1)
R8	200 (1)	13 (1)	2 (1)	0.000132 (−1)	0.000103 (−1)	0.0017 (−1)
R9	0 (−1)	0 (−1)	0 (−1)	0.8 (1)	0.2 (1)	1 (1)
R10	200 (1)	0 (−1)	0 (−1)	0.8 (1)	0.2 (1)	1 (1)
R11	0 (−1)	13 (1)	0 (−1)	0.8 (1)	0.2 (1)	1 (1)
R12	200 (1)	13 (1)	0 (−1)	0.8 (1)	0.2 (1)	1 (1)
R13	0 (−1)	0 (−1)	2 (1)	0.8 (1)	0.2 (1)	1 (1)
R14	200 (1)	0 (−1)	2 (1)	0.8 (1)	0.2 (1)	1 (1)
R15	0 (−1)	13 (1)	2 (1)	0.8 (1)	0.2 (1)	1 (1)
R16	200 (1)	13 (1)	2 (1)	0.8 (1)	0.2 (1)	1 (1)

**Table 2:** **Experimental matrix for central composite design for optimization**

**Run**	**Actual value/coded levels**					**Removed amount of phenanthrene(mg/Kg)**	
	**HA(mg/Kg)**	**Su.(μg/L)**	**Nu. (N , P) (g/L)**				
			**K2HPO4**	**KH2PO4**	**KNO3**	**Experimented value**	**Predicted value**
R1	0(−1)*	5(−1)	0.4(−1)	0.1(−1)	0.5(−1)	196.4	190.67
R2	150(+1)*	5(−1)	0.4(−1)	0.1(−1)	0.5(−1)	222	213.63
R3	0(−1)	13(+1)	0.4(−1)	0.1(−1)	0.5(−1)	208.4	214.23
R4	150(+1)	13(+1)	0.4(−1)	0.1(−1)	0.5(−1)	275.7	274.69
R5	0(−1)	5(−1)	0.8(+1)	0.2(+1)	1(+1)	332.1	331.61
R6	150(+1)	5(−1)	0.8(+1)	0.2(+1)	1(+1)	342.8	335.47
R7	0(−1)	13(+1)	0.8(+1)	0.2(+1)	1(+1)	380.8	387.67
R8	150(+1)	13(+1)	0.8(+1)	0.2(+1)	1(+1)	424.8	429.03
R9	0(−1)	9(0)	0.6(0)	0.15(0)	0.75(0)	247.6	281.05
R10	150(+1)	9(0)	0.6(0)	0.15(0)	0.75(0)	260.8	313.21
R11	75(0)*	5(−1)	0.6(0)	0.15(0)	0.75(0)	283.6	267.85
R12	75(0)	13(+1)	0.6(0)	0.15(0)	0.75(0)	380	326.41
R13	75(0)	9(0)	0.4(−1)	0.1(−1)	0.5(−1)	234.2	223.31
R14	75(0)	9(0)	0.8(+1)	0.2(+1)	1(+1)	394.41	370.95
R15	75(0)	9(0)	0.6(0)	0.15(0)	0.75(0)	340	297.13
R16	75(0)	9(0)	0.6(0)	0.15(0)	0.75(0)	279	297.13
R17	75(0)	9(0)	0.6(0)	0.15(0)	0.75(0)	285.2	297.13
R18	75(0)	9(0)	0.6(0)	0.15(0)	0.75(0)	260.5	297.13

*Low Level: (−1); Middle: (0); High Level: (−1).

### Experimental designs

The experiment was accomplished in two phases; screening of important variables and the levels of them that significantly influenced phenanthrene degradation, followed by optimization of variables levels by using response surface methodology.

#### Screening of variables

Screening step was used for identifying the important of four factors based on full factorial design (2^4^). These relevant factors were Tween 80, as a non-ionic surfactant (Su), HA, Nutrient and Salinity in two levels of high (+1) and low (−1). The importance of the factors was on the base of the largest effect on the biodegradation of phenanthrene in contaminated soil. In this phase 16-run was applied to evaluate factors (variables). Table 1 illustrates the variables and their corresponding levels. The levels of the factors were on the base of previous studies in literature for PAHs bioremediation [14,19-21].

All the experiment runs were performed in triplicates and the average of them was taken as the result. Each of experiment run had the similar chemical control without any inoculation. The statistical software Design Expert V.7, (Stat-Ease, USA) was used to evaluate the analysis of variance (P < 0.05) to determine the significance of each term.

#### Optimization of variables by RSM

The eventual objective of RSM is optimization of the variables by considering the interactive effects of independent factors. With the aim of increasing phenanthrene biodegradation, a central composite face centered design (CCFD) was applied with three factors, which were screened in the first phase. These factors were surfactant, HA and nutrient in 3 levels: High (+1), middle (0) and low (−1) with 4 center points with a CCFD under response surface methodology (RSM) (Table 2). In the first phase salinity had not significant effect on the results, so it was fixed on the 1% W/V.

After running the 18 trials, a second-order polynomial model corresponding to CCFD was fitted to correlate the relationship between the independent variables and the highest percent removal of phenanthrene as a response. The linear computer-generated model is given as [14] (Eq. 1):

1 $$ Y={\beta}_0+{\beta}_1{x}_1+{\beta}_2{x}_2+\dots +{\beta}_k{x}_k+\epsilon $$

Where y is the measured response; β_0_ is an intercept, β_1_ and β_2_ are the linear effect (coefficients computed from the observed experimental values of y) regression terms; x_1_ and x_2_ is the coded level of independent variable; and k indicates the number of independent variables and *є* is the error. The significance of each coefficient in the equation was determined by Student t-test and P-values. Also, F-test indicated all of the factors and interactions considered in the experimental design that are statistically significant (P < 0.05) at the 95 per cent confidence level.

### Determination of microbial population

Most Probable Number (MPN) was used for identification of bacterial population at different time intervals. The bacterial suspension was diluted tenfold serially in a sterile ringer solution (8.5 g NaCl per 1 L DW) and added to the sterile Nutrient Broth in the ratio of 10% of volume in triplicates in five series. After 48 hours of incubation in 30°C, the turbidity of positive growth was seen with direct observation. The population of bacterial consortium was estimated according to statistical table of MPN [6].

### Extraction and analysis

The residual phenanthrene in the soil was extracted with acetone by ultrasonic homogenizer (Bandelin Sonoplus HD 2070) according to EPA 3550B (EPA) for two minutes [22]. The extracted sample was then centrifuged (Hettich D7200) for 15 minutes at 6000 rpm and filtered by Whatman cellulose filter papers N.42 through 2–3 cm of glass wool. A portion of the filtered solution was used for analysis.

The extract was quantified by gas chromatography (GC; Chrompack CP 9001) using a flame ionization detector (FID) with an HP5 capillary column (length of 30 m, inside diameter of 0.32 mm and a coated-film thickness of 0.2 μm). α-Naphthol was used as an internal standard. Initial temperature of the oven was maintained at 100°C for one minute, and then it was increased at a rate of 10°C/min until 250°C. The injector and detector temperatures were set to 250°C and 270°C, respectively. The concentration of phenanthrene was determined after the calibration of the method with standard phenanthrene samples.

## Results and discussion

### Complete factorial design and bioremediation results

**Figure 1:**
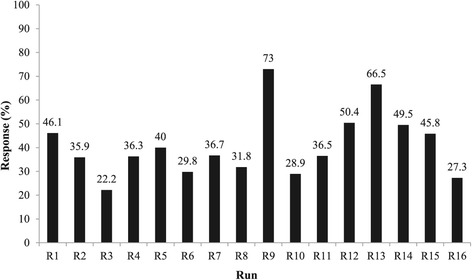
**The phenanthrene removal percentage from Soil in 16 run of complete factorial design samples in comparison to similar controls (Response).**

This stage has attempted to investigate the effect of four independed variables on reducing the phenanthrene level by biodegradation. The phenanthrene removal efficiency in 16 tests using biodegradation compared with their similar control (without inoculation) in 8 weeks was shown in Figure 1 which is about the effectiveness of variables in comparison with each other and Bonferroni line (significant). In these 16 experiments the decrease of phenanthrene concentration in the soil was in the range of 22–73 percent. These results indicate that the addition of microorganisms increased the rate of biodegradation. Our previous study showed that the presence of bacteria in soil has the critical role in the removal of phenanthrene [4,6]. Kästner et al. [23] reported that the biodegradation of pyrene and anthracene had been enhanced by using pure bacterial culture. This increasing was about sixfold in pyrene and tenfold in anthracene removal. The promotion of PAHs mineralization by using mix bacterial culture was reported by Jacques et al. too [24].

After performing 16 runs of complete factorial design, the statistical analysis of the responses was done with regard to the coded design matrix. The responses showed that linear term of HA, Surfactant and Nutrient have remarkable effects on phenanthrene removal efficiency, but salinity was not significant variable in these concentrations. The idea of less effect of salinity is not agree with Chen et al. [7] who reported that salinity was the most significant factor on phenanthrene biodegradation.

**Figure 2:**
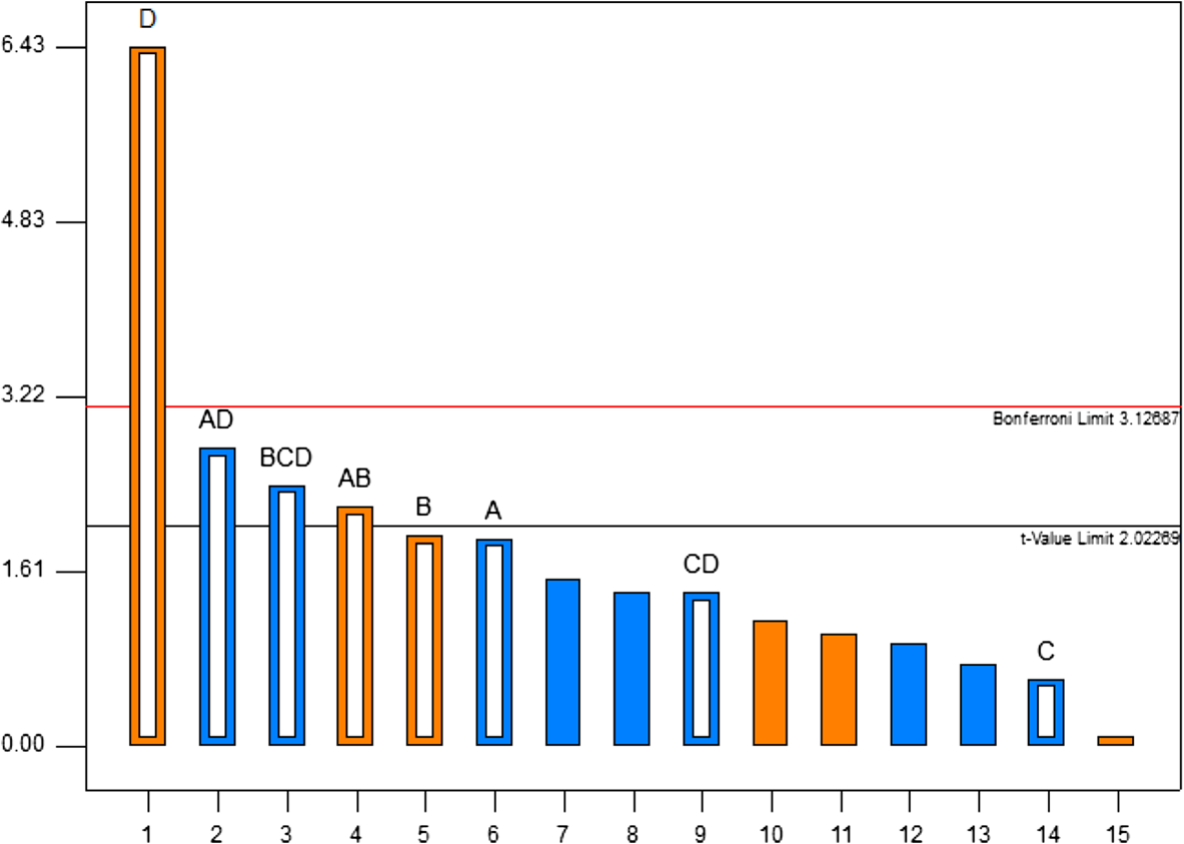
**Standardization (P = 95%) main effects Pareto chart for the complete factorial design: A, HA— humic acid (mg/kg); B, Su—Tween 80 (μL/L); C, Sal— salinity (%W/V); D, Nut— nutrients (g/L), Red line is Bonferroni limitation line.**

Analysis of variance (ANOVA) indicated that nutrient with the largest effect was the most significant variable (p < 0.0001), and HA and Su were possibly significant. The statistically relevant effects were sorted from the largest to the smallest in the Pareto chart presented in Figure 2 that shows the main effect of variables in relation with the slope of the lines. In the Pareto chart all the effect which are in the right the t-value line are significant and each effect which are larger than Bonferroni limitation line have high significant. Hence HA, Su and nutrient were selected as important factors for bioremediation of phenanthrene.

**Figure 3:**
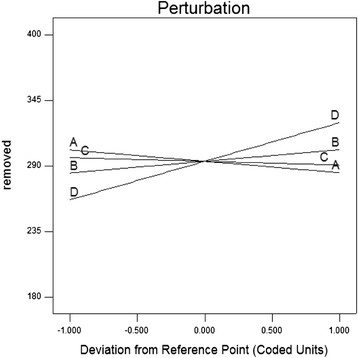
**Perturbation plots for phenanthrene removal (mg/kg) in screening phase: A, HA— humic acid (mg/kg); B, Su—Tween 80 (μL/L); C, Sal— salinity (%W/V); D, Nut— nutrients (g/L).**

Figure 3 shows the statistical fitness between actual and predicted values. The main effect plot (Figure 3) shows the comparative effects of all parameters on biodegradation of phenanthrene. The slope of nutrient effect shows that the response of phenanthrene removal was sensitive to this factor. The relatively flat line of salinity shows insensitivity of the responses to change in this parameter. The slope of other two parameters confirmed the possibly significant role in the process. Betancur-Galvis et al. [12] reported that the concentration of phenanthrene was 1.7-times and 2.9-times lower in the soil amended with NP and biosolid respectively compared to sterilized soil. In our study application of high level of N and P in run 9 had been increased the phenanthrene removal by more than 1.5 fold compared to low level of N and P in run 1. Da Silva et al. [25] showed that bioremediation of coastal sand contaminated crude oil would be improved through biostimulation by using commercial mineral NPK fertilizer.

### Optimization and verification

The structural matrix using three factors CCFD with 4 center points, experimental and predicted responses were presented in Table 2.

In order to explain the effect of each variable and their interactions on the phenanthrene removal, the first-order model was given by the following equation (Eq.2):

2 $$ \begin{array}{l} Removed\  phenanthrene\left( mg/ kg\right)=297.13+16.08A+29.28B+73.82C\\ {}+9.38 AB-4.77 AC+8.13BC\end{array} $$

**Figure 4:**
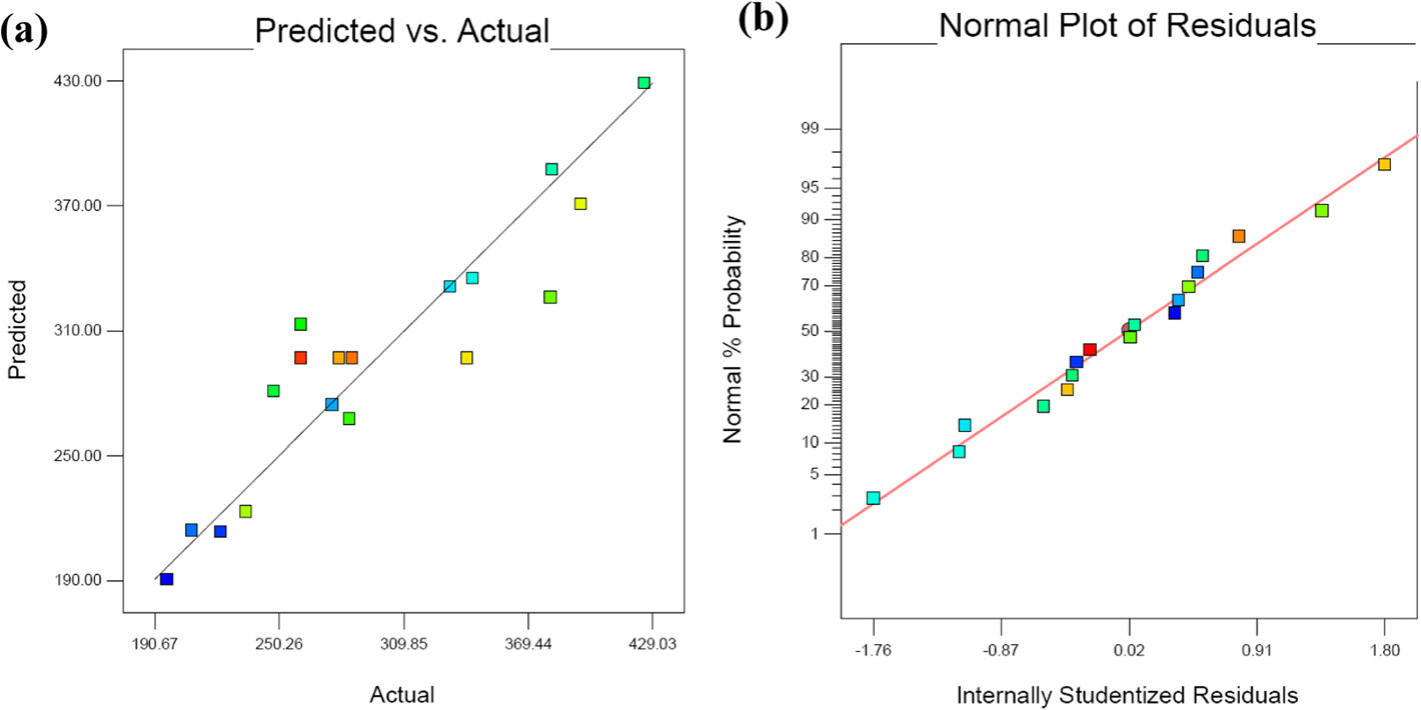
**(a) Predicted versus actual and (b) Normal plot of phenanthrene in optimization phase.**

**Figure 5:**
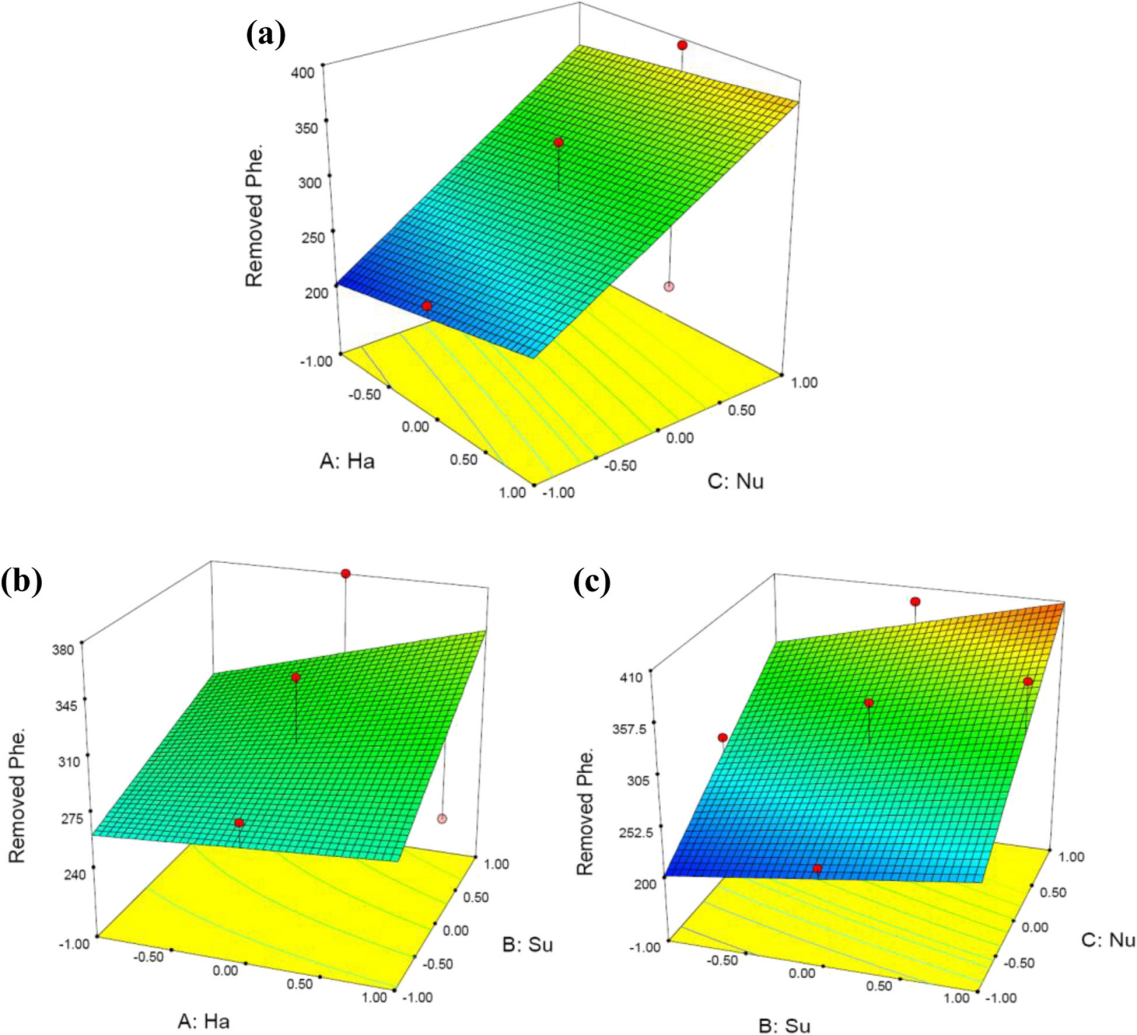
**Response surface plot for phenanthrene removal as function of; (a) HA and nutrient, (b) HA and surfactant, (c) Nutrient and surfactant interactions.**

Where A is HA concentration, B is Surfactant concentration and C is the concentrations of Nutrient (as N and P). The model was examined by the regression equation and determination coefficient (R^2^ = 0.883) which suggests that more than 88.3 percent of the variance is attributable to the variables (Figure 4). The F-value of the regression model was 10.63, which implied that the application of linear model was satisfactory for the assessment of phenanthrene biodegradation in the soil. Also the low probability value (<0.0005) indicated a high significance of the model. Among the independed variables the linear term of surfactant had significant effect (P < 0.05), while the linear term of nutrient had highly significant effect (P < 0.0001) on phenanthrene removal. All the parameters and their interaction had positive effect except the interaction of HA and nutrient had negative effect, which indicated that increasing their levels would decrease the biodegradation of phenanthrene. Figure 5 represent three-dimensional plot of the variables. The parallel contours in Figure 5a,b and c, respectively suggested that interactions of HA to nutrient, HA to surfactant and surfactant to nutrient could be negligible that confirmed by the percent of their effects which was <1%.

With emphasis on nutrient effect, lower HA values together with higher nutrient levels or higher HA values together with lower nutrient levels could result in higher biodegradation values. Very slight variations in phenanthrene removal efficiency (Figure 5a and b) were obtained when the HA was used up to concentration of 150 mg/kg soil corresponding to surfactant and nutrient from low level to high level. The F-ratio of 3.85% had confirmed it too. In spite of increasing the solubility of PAH in the presence of humic substance [26,27], HA had no significant effect on phenanthrene biodegradation and it is agree with Heywood et al. [28] who reported that there was not any relationship between PAHs biodegradation and the organic matter content of the soil. The bond between the contaminants and HA can make the contaminants to be more resistant against desorption and biodegradation by microorganisms [29]. Macleod and Semple and Semple et al. reported that in the soil containing humic compounds the mineralization of pyrene occurred with a retardation which was attributed to the slow desorption of HOCs (Hydrophobic Organic Contaminants) as a rate-limiting factor in biodegradation [15,30]. In our previous study in presence of humic compounds, retardation in phenanthrene biodegradation was seen too [6].

The analysis of variance shows that the effect of surfactant on phenanthrene biodegradation was more than HA and less than nutrient. Its middle effect was shown in 3D plot too (Figure 5a and b). Mixed results have been reported concerning the effect on the biodegradation of PAHs by the addition of surfactant. The positive effect of surfactant is enhancement of biodegradation via increasing in solubility of phenanthrene in water phase which may lead to be more bioavailable for degrading microorganisms [8,31]. The negative effect of surfactant has been attributed to be used by microorganisms as a preferential growth substrate or may be toxic for microorganisms because of high amount of contaminant which have been soluble [8]. The lower amount of MPN may be related to high concentration of phenanthrene in liquid phase (data was not shown). The bacterial cell lyses was reported by Avramova et al. [8] in prevention of phenanthrene mineralization by *Pseudomonas sp*. In their research addition of Triton X-100 inhibited the phenanthrene mineralization too. Piskonen and Itävaara [21] showed that using four types of surfactant at lower concentrations of CMC could not enhance the PHAs bioremediation in contaminated soils.

In Figure 3, a steep slope of in nutrient curve shows that the response of phenanthrene removal was very sensitive to this factor. The effect of this factor was the highest in second phase of experiment too, which was confirmed by ANOVA. Figure 5a and c. show that nutrient is a crucial factor for the biodegradation of phenanthrene. A highly variation of hydrocarbon biodegradation in the same aquifer samples with nutrient addition was reported in the other research too. Breedveld and Sparrevik [32] observed a strong relation between biodegradation of fluoranthene with nitrogen and phosphorous content. They reported that the transformation rates of hydrocarbons have been increased by addition of inorganic nitrogen and phosphorous. Similar conclusion was also drawn from Coulon et al. [33] who showed that the addition of a commercial oleophilic fertilizer containing N and P had a positive effect on degradation of TPH in contaminated sub-Antarctic soil. But it is not agree with Chen et al. [7] who reported that nutrient was the insignificant factor on phenanthrene biodegradation [8].

Enhancement in biodegradation rates of oil-contaminated sediments with addition of inorganic nutrients had been reported before [34]. Nutrient comprises nitrogen, phosphorous and other inorganic elements are essential for bacterial growth [11] and addition of them can enhance bacterial activity, which causes in biodegradation of pollutant [12]. Similar to our research, Mohajeri et al. [35] reported that the higher dose of nutrient amendment can accelerate the phenanthrene biodegradation rate.

The predicted values of phenanthrene removal were calculated from the first-order model and the actual values were determined for particular runs. The predicted versus actual plot of phenanthrene biodegradation and normal percent probability versus the studentized residuals plot were shown in Figure 4a and b. the similarity between the observed values of experiment and the fitted values under the suggested model indicate that model prediction is accurate. The studentized residuals illustrate the normal distribution, the goodness of fit and linearity of the fitted model.

According to the results, the highest biodegradation (85% = 424.8 mg/kg) was obtained from run8 (150 mg HA/Kg soil, 13 μg/L surfactant, and nutrients as K_2_HPO_4_: 0.8; KH_2_PO_4_: 0.2; KNO_3_: 1 g/L). In this phase the important sequence of the factors was Nutrient, Surfactant and HA with the effect of 81.27%, 12.78% and 3.58%, respectively.

CCFD method predicted that the removal of phenanthrene under the optimum conditions (150 mg HA/Kg soil, 12.68 μg/L surfactant, and nutrients as K_2_HPO_4_: 0.8; KH_2_PO_4_: 0.2; KNO_3_: 1 g/L) will be 87.1%. The average (n = 5) of the obtained percent removal of phenanthrene in synthetic contaminated soil under the optimum conditions was 85.3 ± 1.5%. The potential application of optimum conditions resulted from the model was tested by determining the phenanthrene removal from the real contaminated soil samples. The contaminated soil samples were obtained near the Tehran Petroleum Refinery. The compositions of soil samples were consisted of 69% sand, 10.0% silt and 21% clay and the phenanthrene concentration was in the range of 32–45 mg/kg. The average (n = 5) removal of phenanthrene in contaminated soil samples under the optimum conditions was 72.2 ± 5.8%. Based on the established results, there are satisfactory agreements between the results for the estimated responses and those obtained under the optimum conditions.

The highest biodegradation rate of phenanthrene in optimum condition was 7.78 mg phenanthrene per kg soil per day; whereas the phenanthrene biodegradation rate in lowest amount of these factors were only 3.5078 mg phenanthrene per kg soil per day. Data of this research showed that optimization of effective factors in bioremediation of PAHs contaminated soil could increase the biodegradation rate by two fold of magnitude.

## Conclusions

The screening experiments showed that significant factors in phenanthrene biodegradation were HA, surfactant and nutrient contents. Therefore, these variables were used for RSM. The obtained results from RSM, point out the importance of nutrients for phenanthrene biodegradation. Surfactant displayed significant positive effect and HA had an insignificant effect on phenanthrene removal. A high similarity was between the model prediction and experimental results. According to the effective ratio, the sequence importance of the variables on phenanthrene biodegradation in contaminated soils were as Nutrient > Surfactant > Humic acid > Salinity. The biodegradation rate at optimum condition was 7.78 mg phenanthrene/kg soil/day.
